# The effect of neutrophil extracellular traps in venous thrombosis

**DOI:** 10.1186/s12959-023-00512-4

**Published:** 2023-06-16

**Authors:** Weiwei Li, Zixiang Wang, Chen’guang Su, Zheng Liao, Yinxuan Pei, Jianli Wang, Zixin Li, Shijie Fu, Jinlong Liu

**Affiliations:** 1 Department of Hepatobiliary Surgery, Affiliated Hospital of Chengde Medical University, Chengde, Hebei Province 067000 China; 2 Department of Pathology, Affiliated Hospital of Chengde Medical University, Chengde, Hebei Province 067000 China; 3 Department of Orthopedic, Affiliated Hospital of Chengde Medical University, Chengde, Hebei Province 067000 China

**Keywords:** Venous thrombosis, Neutrophil, Neutrophil extracellular traps, Humans

## Abstract

Neutrophil extracellular traps (NETs) as special release products of neutrophils have received extensive attention. They are composed of decondensed chromatin and coated with nucleoproteins, including histones and some granulosa proteins. NETs can form a network structure to effectively capture and eliminate pathogens and prevent their spread. Not only that, recent studies have shown that NETs also play an important role in venous thrombosis. This review provides the most important updated evidence regarding the mechanism of NETs formation and the role of NETs in the process of venous thrombosis. The potential prophylactic and therapeutic value of NETs in venous thrombotic disease will also be discussed.

## Introduction

More recently, it has been recognized that the release of neutrophil extracellular traps (NETs) may lead to venous thrombosis. NETs are primarily caused by stimulated neutrophils and were first described by Brinkmann et al. in 2004 [1]. They are composed of decondensed chromatin and coated with nucleoproteins, including histones and granulosa proteins such as neutrophil elastase (NE) and myeloperoxidase (MPO), forming a network DNA structure [2, 3]. This review focuses on the role of NETs and their main components in venous thromboembolism (VTE), and explores the potential therapeutic significance of NETs.

### Formation of NETs

**Fig. 1:**
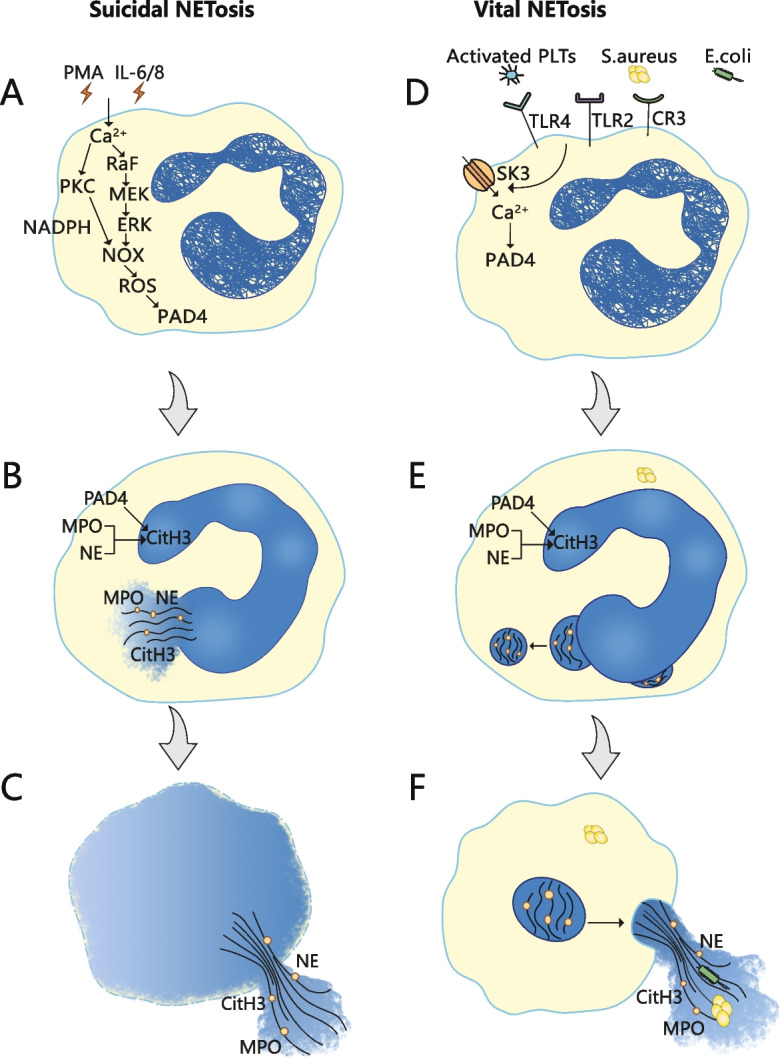
Two types of process of NETosis: Suicidal NETosis (**A**-**C**) vs vital NETosis (**D**-**F**). **A** Various substances, including PMA and cytokines, can induce suicidal NETosis. Subsequent increases in cytoplasmic calcium activate PKC or Raf/MEK/ERK kinase pathways, which in turn activate niacinamide adenine dinucleotide phosphate (NADPH) oxidase complexes (NOX) and subsequent reactive oxygen species (ROS) production. **B** Peptidyl arginine deiminase-4 (PAD4), together with neutrophil elastase (NE) and myeloperoxidase (MPO), induces histone H3 citcitylation (CitH3), which further leads to decondensation of chromatin and loss of the lobular shape of the nucleus. Subsequently, nuclear envelope rupture and chromatin expansion into the cytoplasm. **C** Plasma membrane rupture and NET release. **D** In contrast, vital NETosis can be stimulated by activated platelets (PLTs), microorganisms, and complement proteins. And then, toll-like receptors (TLR) 2 and TLR 4 can be activated, leading to the activation of PAD4. Small conductance potassium channel member 3 (SK3) activated by calcium is also involved in the regulation. **E** Decondensation of chromatin and loss of the lobular shape of the nucleus. The external and internal nuclear membranes are then separated and the vesicles sprout. **F** NETs are sent out of the neutrophil by vesicles. This process protects the outer membrane of the neutrophils, thus allowing them to continue to function, even to the point of becoming anuclear

In contrast to normal apoptosis and necrosis, neutrophils undergo a carefully planned cell death pattern to release NETs, a process known as NETosis [4]. The formation of NETs can be divided into suicidal NETosis and vital NETosis depending on nature of the stimulation, activation of signaling pathways, and cell membrane integrity [5, 6] (Fig. 1). Suicidal NETosis was primarily discovered following in the context of phorbol 12-myristate 13-acetate (PMA) chemical stimulation. And then, activation of neutrophils can lead to an increase in intracellular calcium concentration. Some kinases activated downstream of calcium influx [7] and cell cycle regulators, such as PKC [8], Raf-MEK-ERK [9, 10] pathways, are all associated with suicidal NETosis. The production of reactive oxygen species (ROS) from nicotinamide adenine dinucleotide phosphate (NADPH) oxidase and mitochondria in neutrophils are also integral to suicidal NETosis induction. Unlike suicidal NETosis, vital NETosis occured without NADPH oxidase activity. Vital NETosis can be stimulated by activated platelets, microorganisms, and complement proteins. It has been shown that during vital NETosis, neutrophils activate toll-like receptors (TLR) 2 and TLR 4 under the stimulation of lipopolysaccharide and gram-negative bacteria, leading to peptidyl arginine deiminase-4 (PAD4) activation. Calcium-activated small conductance potassium channel member three (SK3) was also involved in the regulation [11]. Through this series of signal conduction events, NETosis will be transformed from signal changes to the following morphological changes [12].

Chromatin decondensation is the dominant feature of NETosis. There are two main theories. Most studies suggested that chromatin decondensation in NETosis is mediated by posttranslational modification of histones. PAD4 -mediated citrullination plays a key role in this process [13]. PAD4-mediated citrullination can induce a reduction in histone positive charge. By reducing the affinity between histones and negatively charged DNA, it causes histones to dissociate from DNA, resulting in loss and decondensation of dense chromatin structure [12]. Li et al. [14] showed that PAD4(-/-) neutrophils cannot form NETs when incubated with bacteria, and lack the bactericidal ability of NETs. PAD4 has also been shown to play an important role in NETs formation during aseptic inflammation [15].

Although there is much evidence that PAD4 is associated with the formation of NETs, some studies have taken different views. Kenny et al. [8] demonstrated the formation of NETs in the absence of histone H3 citrullination. Some proteases, such as NE, proteinase 3 (PR3), cathepsin G, and calpains, have all been implicated in NETosis and chromatin decondensation [12, 16]. The main mechanism may be that proteases (especially NE) mediate chromatin decondensation through histone cleavage [17]. However, these views have also been challenged. Martinod et al. [18] showed that NE deficiency did not prevent NETosis and deep vein thrombosis (DVT) results in vivo. Neutrophils from NE-deficient mice still developed NETosis under PMA or platelet-activating factor (PAF) stimulation. Some other studies have found that neutrophils from patients with MPO defects fail to release NETs when stimulated by PMA, Candida albicans, or Group B Streptococcus [8]. Although MPO does not decondense chromatin directly, it has been shown to enhance the process of NE-mediated chromatin decondensation [19]. It seems that NE-mediated chromatin decondensation has a more complex pathway.

Nuelear membrane disintegration and cell lysis were often key factors in the release of NETs in suicidal NETosis. Gasdermin D (GSDMD), a pore-forming protein, could form pores in the plasma membrane and chromatin can be released into the environment. Due to electrostatic interactions, the proteins released from the granules bind tightly to the decondensed chromatin to form NETs [20, 21]. However, GSDMD is not the only pore-forming protein involved in the release of NETs. Some studies have reported that the activation of another pore-forming protein, mixed lineage kinase domain‐like (MLKL) can also lead to the release of NETs [22, 23]. Interestingly, vital NETosis was thought to occur in the absence of rupture of the nuclear envelope. It has been demonstrated that DNA vesicles germinate from the nuclear membrane, cross the cytoplasm, and bind to the plasma membrane, thus delivering the NETs from the cell without the need to cross the membrane. In suicidal NETosis, neutrophil cell membranes rupture so that they can no longer perform recruitment, chemotaxis, and phagocytosis, leading to loss of traditional functions. In contrast, the vital NETosis, a non-suicidal pathway of NETosis, allows NETs release and regular host defense to coexist [24]. Thus, it can be seen that NETosis is not a single and simple process, but involves a variety of mechanisms that may overlap and are still poorly understood, which still require further study.

### NETs and specific conditions of high risk of thrombosis

The development of NETs also varies with different conditions of high risk of thrombosis. When tissue or organ damage occurs, including trauma and ischemia–reperfusion (IR) induced damage, severe inflammatory responses can be induced, and increasing the risk of sepsis. NETs are produced in the context of neutrophil antimicrobial mechanisms. However, the production of NETs may be a double-edged sword [25]. Su et al. [26] showed that activated platelets could promote the formation of NETs and kill pathogens in the early stages of sepsis. However, with the progression of sepsis and the occurrence of platelet pyroptosis, the excess formation of NETs in turn intensifies the inflammatory response and further induces platelet pyroptosis. The latest study found that activation of platelet-specific STING appears to be a critical driver. In the absence of STING, platelet activation and granule secretion were inhibited, thereby alleviating intravascular thrombosis and NETosis in sepsis mice [27]. Moreover, endogenous mitochondrial DNA (mtDNA) and oxidized mtDNA were observed to induce the formation of NETs and sterile inflammation in acute peripheral tissue trauma models [28].

Recent studies have also reported a strong relationship between NETs and immune thrombosis in patients with COVID-19, and have identified the development of cytokine storms in COVID-19 patients [29, 30]. Dysregulated activation of a variety of leukocytes (including neutrophils, monocytes, macrophages, B-lymphocytes and T-lymphocytes, etc.) and epithelial or endothelial cells induces cytokine storms that release large amounts of pro-inflammatory cytokines. The above environment also plays an important role in the activation of NETosis, and the NETosis process is induced by cytokines, chemokines, antibodies and immune complexes.

In addition to the factors mentioned above, other factors such as pregnancy and malignancy have been reported to be associated with an increased risk of VTE. Giaglis et al. [31] have reported that maternal obstetric neutrophils exhibit a strong pro-NETotic phenotype with increased CitH3, MPO, NE, and ROS plasma levels, compared to non-pregnant and pregnant controls, and are associated with an increased risk of thrombosis. Unlike other NETs formation, NETs formation is primarily driven by G-CSF and finely regulated by sex hormones in the context of the systemic milieu formed during pregnancy [32].

Additionally, complex interactions between malignancy, inflammation, and hemostatic systems are increasingly being extensively studied. Abdol et al. [33] found that rapid formation of NETs was induced when human pancreatic cancer cells (AsPC-1) were co-cultured with neutrophils isolated from healthy individuals. Moreover, ASPC-1-activated platelets promoted the release of NETs compared with unstimulated platelets. Yang et al. [34] found that neutrophils isolated from patients with gastric cancer had a greater ability to spontaneously form NETs than neutrophils isolated from healthy individuals. Li et al. [35] found similar results that NETs were more likely to form in blood and tissue samples from patients with gastric cancer than in healthy individuals. These data suggest that malignancy-specific environments may be associated with systemic changes in neutrophils and NETs.

Recent studies have raised interest in low-density neutrophils (LDNs). As a subpopulation of neutrophils, LDNs are increased in severe infections [36, 37], COVID-19 [38] and malignancy [39], etc. Although LDNs display decreased ability to phagocytose bacteria, their ability to form NETs was significantly enhanced [40]. LDNs have proved to be the subpopulation of neutrophils prone to NETs production compared to other cell types. All in all, the results of various studies show that the formation of NETs exhibits different characteristics under specific condition or tissue, which has important research significance.

### The role of NETs in promoting thrombosis

The thrombogenic effect of NETs was first proposed by Fuchs et al. in 2010 [41]. NETs can form a histone-DNA reticular scaffold to capture platelets, erythrocytes and other substances, such as fibrinogen, von Willebrand factor (VWF), tissue factor (TF) and coagulation factor XII (FXII), which could conducive to thrombosis [42]. The network structure of NETs not only forms the structural basis of thrombus, but also can increase the stability of fibrin scaffold in thrombus. Some studies have reported that citrullinated fibrinogen can decrease fiber diameter and porosity, increase the density of fibrin, and reduce the solubility of fibrin clots [43]. And fibrous proteins containing histone-DNA complexes have thicker fibrin fibers, greater stability and rigidity. This combination significantly prolongs the clot dissolution time [44].

In addition, some other components of NETs can also promote platelet activation and aggregation, facilitating the formation of thrombus. Histones in NETs can activate platelets by utilizing TLR 2 and TLR 4, and further enhance platelet aggregation and activation through recruitment of fibrinogen to promote thrombin production [45, 46]. Histones can also stimulate the release of VWF from vascular endothelial cells, further mediating platelet adhesion and aggregation [47]. Meanwhile, histones can promote the expression of TF in vascular endothelial cells and monocytes, and initiate activating external coagulation pathways. The coagulation pathways induced by FXII and TF can also be further enhanced by NE and cathepsin G through hydrolyzed tissue factor pathway inhibitor (TFPI) [45, 48]. NETs can also directly bind FXII and further enhance the activation of FXIIa in coordination with platelets [49, 50].

Some studies suggested that NETs play a role primarily in the early stages of thrombosis. de Boer et al. [51] found that NETs were mainly present in fresh and lytic, but not in organized thrombus. Savchenko et al. [52] analyzed thrombus samples obtained during surgery or at autopsy and showed that NETosis occurs in the organizing stage of thrombus development. Moreover, NETs markers such as CitH3 (citrullinated histones 3) were different at each stage of thrombosis, and they were replaced by collagen fibers later in thrombus formation. Sharma et al. [53] suggested that neutrophil inflammation and NETs also play an important role in chronic thrombosis. NETs and their components mediate fibrotic remodeling of thrombus by enhancing transforming growth factor-β (TGF-β) signaling and promoting differentiation of monocytes into activated fibroblasts. There are indications that NETs appear to play different roles in different stages of thrombus formation.

Many studies have described the direct and indirect mechanisms by which NETs promote thrombosis. However, the vast majority of these studies researched components of NETs rather than intact NETs. Noubouossie et al. [54] questioned the ability of intact NETs to directly activate coagulation. Compared with intact NETs and nucleosomes, purified DNA and histones were observed to induce thrombin production more effectively. It is possible that the tight packing of histones and DNA in the nucleosome partially reduces their ability to interact with the clotting system. Although the relative contribution of intact NETs and their individual NETs components to activating coagulation remains controversial, it is clear that NETs can influence the development of thrombosis in a variety of ways, and the evidences are also emerging about how NETs promote thrombosis.

### Biomarkers of NETs in venous thrombosis related diseases

The presence of NETs or their key components such as extracellular DNA, nucleosomes, MPO, CitH3, or NE in human venous thrombosis related diseases has also been further investigated. Mauracher et al. [55] showed that elevated plasma levels of cell-free DNA (cfDNA) and nucleosomes appeared to predict VTE in the short term. In other venous thrombotic diseases, such as DVT [56–58], pulmonary embolism (PE) [59], portal vein thrombosis (PVT) [60, 61], and cerebral venous sinus thrombosis (CVST) [62], high plasma levels of NETs have been shown to be associated with increased risks of thrombosis. By analyzing organizing thrombi obtained during surgery or autopsy, several studies supported the role of key components of NETs in venous thrombosis and stabilization [52, 62, 63]. Moreover, Ząbczyk et al. [59] observed that elevated circulating CitH3 may be linked to a higher early mortality risk in patients with acute PE. NETs or their key components showed potential diagnostic and prognostic value as biomarkers in venous thrombosis related diseases.

NETs can be visualized at high resolution by a scanning or transmission electron microscope [64], or tissue immunoassay of granule proteins bound to DNA or histones by a conventional microscope. In order to exclude interference from extracellular DNA and granulosa proteins sources other than NETs, multiple staining of NETs components, such as extracellular DNA, nucleosomes, MPO, CitH3 and NE, is recommended for colocalization [65, 66]. Of course, morphological standards of microscopic observation are not quantitative [67]. Nevertheless, the clinical application of NETs or their key components as biomarkers of thrombosis remains problematic. As tissue samples are often difficult to obtain in clinical practice, key NETs components are usually tested using blood samples, such as enzyme-linked immunosorbent assays (Elisa), which has gradually become one of the most widely used clinical methods for NETs. But unfortunately, Elisa provides relatively limited information, due to lack of specificity and standardized testing [68, 69]. Other methods, such as the use of flow cytometry to detect NETs components on cells [70] or extracellular vesicles (EVs) [71], are also being considered as novel methods to assess NETs formation in vivo. But again, the lack of objectivity and specificity of these methods remains the problems that need to be addressed. Therefore, appropriate assays specifically for the measurement of NETs or their components will be worthy of further exploration in future studies.

### Potential prophylactic and therapeutic value of NETs in venous thrombosis

Since NETs play an important role in thrombosis, they provide a potential target for the prophylaxis and treatment of venous thrombotic diseases. At present, most researches focus on promoting the degradation and inhibiting the formation of NETs.

#### Promotion of NETs degradation

Due to the essence of NETs, which are high degree of decondensed chromatin in the extracellular environment, deoxyribonuclease (DNase) seems to become a promising targeted prophylaxis and treatment strategy for NETs. Two major DNases had been identified, including DNase 1 and DNase 1-like 3 (DNase 1-L3) [72]. Since DNase1 and DNase 1-L3 are expressed independently, they can provide dual host protection and are effective against harmful effects of intravascular NETs [72]. At present, most research directions were focused on the application of DNase 1 in NETs. Treatment of plasma with DNase 1 has been shown to reverse the increase in thrombin generation and protects from venous thrombosis during aging [73]. Jimenez-Alcazar et al. [74] reported that plasma levels of DNase 1 were reduced in patients with acute thrombotic microangiopathy (TMA), and NETs degradation activity was restored by recombinant human DNase 1 supplementation. This suggests that the restoration of plasma DNase 1 activity may be the potential prevention and treatment measures.

DNase 1 in combination with other medicines seems to have unexpected effects. In vitro experiments, the combination of DNase 1 with tissue plasminogen activator (tPA) could accelerate thrombolysis when the DNase 1 alone was ineffective [75]. Other studies have also shown that DNase 1 combined with tPA can achieve complete thrombolysis, making up for the defects of incomplete and inefficient thrombolysis of tPA alone, and may improve the therapeutic window of thrombotic disease [63, 76]. Manfredi et al. [77] reported that the treatment of low molecular weight heparins (LMWH) could abrogate the ability of neutrophils to generate NETs and neutralize the harmful effects of histones. Heparins combination with DNase 1 may further reduce the risk of future thrombotic events. The interaction between NETs and VWF also contributes to venous thrombosis. Recombinant ADAMTS13 (rADAMTS13) has been shown to prevent thrombosis by reducing initial platelet accumulation and neutrophil recruitment [78]. Targeting the NET-VWF axis via a combination of DNase 1 and ADAMTS13 has also been shown to be a potential therapeutic approach [78].

Although DNase has shown strong potential in the prophylaxis and treatment of venous thrombotic disease alone or in combination with other medicines, there are still some concerns in this regard. In some cases, treatments that use DNase 1 to lysate NETs that have been formed and released may result in the rapid release of the contents of the NETs (such as histones and MPO) or the microorganisms that have accumulated in the NETs, which then promote further inflammation and thrombosis. Since the effects between NETs and thrombosis may be reciprocal, this may put patients with thrombosis at an increased risk of thrombosis after infection [79]. The efficacy and safety of DNase 1 still remain to be tested and further studies are needed.

#### Inhibition of the formation of NETs

The PADs are a family of enzymes (including PAD1, 2, 3, 4, and 6) that can convert protein arginine residues to produce citrulline. Among them, PAD4 is considered to play an essential role in the formation of NETs [80, 81]. PAD4 deficiency has been shown to reduce the occurrence of thrombosis in a mouse model of venous thrombosis. And importantly,in this model, PAD4 deficiency did not lead to increased susceptibility to bacterial infection, which may indicate that NET deficiency due to PAD4 inhibition does not result in a significantly impaired host immune function [82]. Several PAD inhibitors, such as F- and Cl-amidine [83], have been shown to be effective against PAD4 under different conditions. However, because they inhibit several other subtypes of PAD at the same time, these PAD inhibitors lack the specificity required for clinical treatment. Recently, reversible PAD inhibitors of GSK199 and GSK484 have been developed that show high specificity against PAD4 and can effectively inhibit the formation of NETs in mice and human neutrophils [84]. But further studies are needed to verify its therapeutic potential for vein thrombosis.

ROS also plays an important role in the formation of NETs. Currently, N-acetylcysteine (NAC) has been shown the efficient antioxidant activity in a variety of ROS-related diseases [85], and can inhibit formation of NETs by inhibiting the production of ROS [86]. Other antioxidants, such as vitamin C, also known as ascorbic acid, have also been shown to reduce the formation of NETs in sepsis [87] and can effectively reduce the mortality of patients with sepsis [88]. Recent studies have suggested that molecular hydrogen (H_2_) therapy, by inhaling H_2_, can effectively inhibit neutrophils activation and excess NETs formation without inhibiting the essential function of neutrophils, and H_2_ appears to be more effective than other antioxidants, such as NAC or ascorbic acid [89].

As the key components of NETs, NE and CitH3 have also become potential prophylactic and therapeutic targets for NET-related anti-thrombotic therapy. Several studies have shown that small molecule inhibitors of NE, such as sivelestat [90] and CitH3 inhibitors, such as therapeutic anti-citrullinated protein antibody (tACPA) [91], can effectively inhibit the formation of NETs in mice and humans. More recently, neonatal NET inhibitory factor (nNIF), a novel NETosis inhibitor, has been shown to inhibit key terminal events in NETs formation, including histone H3 citrullination and the activity of PAD4 [92]. In addition to the above drugs, there are some other substances that may inhibit the formation of NETs, such as anti-P-selectin aptamer or anti-P-selectin glycoprotein ligand-1 (PSGL-1) [93], aspirin [94], vitamin D [95], chloroquine [96, 97], metformin [98], etc. But the mechanisms of these findings may remain unclear.

In conclusion, promotion of NETs degradation or inhibition of the formation of NETs have the potential to prevent and treat venous thrombosis in diseases with high risk of thrombosis such as trauma, sepsis, pregnancy, COVID-19 and malignancy, etc. Although the specific effects on venous thrombosis need to be further validated, they represent possible different directions for future research.

### Conclusion

At present, NETs or their key components, such as extracellular DNA, nucleosome, MPO, CitH3 and NE, have been shown to play a role in venous thrombosis in a large number of clinical samples and animal experiments, showing their potential as biomarkers to provide evidence for the diagnosis or prognosis of venous thrombosis related diseases. However, it should be noted that the sensitivity of NETs or their components still needs to be further improved and better and more accurate measurements are needed. Meanwhile, according to the significant role of NETs in promoting thrombosis, it also provides various potential directions for the prophylaxis and treatment of venous thrombotic diseases, which is worthy of further extensive research. In conclusion, as a unique immune mechanism after neutrophil death, the unique role of NETs in promoting venous thrombosis is of great significance for the further diagnosis and treatment of venous thrombotic diseases.

## Data Availability

Not applicable.
